# Tolerance Induction by Exosomes from Immature Dendritic Cells and Rapamycin in a Mouse Cardiac Allograft Model

**DOI:** 10.1371/journal.pone.0044045

**Published:** 2012-08-29

**Authors:** Xiao Li, Jun-Jie Li, Jing-Yue Yang, De-Sheng Wang, Wei Zhao, Wen-Jie Song, Wei-Min Li, Jian-Feng Wang, Wei Han, Zhuo-Chao Zhang, Yong Yu, Da-Yong Cao, Ke-Feng Dou

**Affiliations:** 1 Department of Hepatobiliary Surgery, Xijing Hospital, Fourth Military Medical University, Xi’an, Shaanxi Province, China; 2 Department of Clinical Oncology, Xijing Hospital, Fourth Military Medical University, Xi’an, Shaanxi Province, China; MRC National Institute for Medical Research, United Kingdom

## Abstract

**Background:**

Dendritic cells (DCs) release bioactive exosomes that play an important role in immune regulation. Because they express low levels of class I major histocompatibility complex (MHC) and co-stimulatory molecules, exosomes derived from donor immature DCs (imDex) prolong allograft survival by inhibiting T-cell activation. However, this effect is limited and does not induce immunological tolerance when imDex are administered alone. Thus, we tested the effect of combined treatment with donor imDex and low-dose rapamycin on inducing tolerance in a mouse cardiac transplantation model.

**Methods:**

ImDex were obtained from the culture supernatant of immature DCs derived from donor mouse (C57BL/6) bone marrow and were injected with suboptimal doses of rapamycin into recipient mouse (BALB/c) before and after transplantation. The capacity of this treatment to induce immune tolerance was analyzed in vitro and in vivo using the mouse cardiac transplantation model.

**Results:**

Donor imDex expressed moderate levels of MHC class II and low levels of MHC class I and co-stimulatory molecules, but neither imDex nor subtherapeutic rapamycin dose alone induced cardiac allograft tolerance. Combined treatment with imDex and rapamycin, however, led to donor specific cardiac allograft tolerance. This effect was accompanied by decreased anti-donor antigen cellular response and an increased percentage of spleen CD4^+^CD25^+^ T cells in recipients. Furthermore, this donor specific tolerance could be further transferred to naïve allograft recipients through injection of splenocytes, but not serum, from tolerant recipients.

**Conclusion:**

Combined with immunosuppressive treatment, donor imDex can prolong cardiac allograft survival and induce donor specific allograft tolerance.

## Introduction

Organ transplantation is almost the only hope of complete cure for patients with organ failure. In addition to organ shortage, immune rejection is the biggest obstacle to the development of organ transplantation [1]. Although non-specific immunosuppressants can suppress immune rejection and prolong allograft survival, long-term use of these drugs causes serious adverse reactions, such as increased occurrence of opportunistic infections or increased cancer recurrence rate [2]. Thus, donor specific tolerance must be established and maintained to reduce the dosage of immunosuppressants [3].

Dendritic cells (DCs) are the professional antigen-presenting cells (APCs) that present donor alloantigen to recipient T cells. The view that immature and mature DCs mediate different functional T-cell responses (i.e., tolerance versus priming) is very common, and the effect of immature DCs (imDCs) on inducing transplantation tolerance has been tested in many different animal transplantation models. However, this DC-based technology has some deficiencies that limit its application in the clinic, including potential maturation and short-term lifetime in vivo, the requirement of 7 days (d) for production, and short-term preservation in vitro [4], [5]. Thus, donor imDCs cannot be used repeatedly and do not induce sufficient immune tolerance.

Exosomes are small membrane vesicles (diameter 50–100 nm) of late endocytic compartment origin secreted by a variety of cell types [6]. DC-derived exosomes (Dex) present antigen-major histocompatibility complex (MHC) and co-stimulatory molecules to T lymphocytes and therefore have strong immunological regulatory activities [7], [8]. Depending on the maturation state of DCs producing exosomes, Dex induces T-cell tolerance or priming. Mature DC-derived exosomes (mDex) trigger effector T-cell response and lead to fast skin allograft rejection [9], whereas imDC-derived exosomes (imDex) display a certain degree of immunosuppressive activity in autoimmune diseases [10]–[12] and animal models of allogenetic organ transplantation [13]–[15]. Moreover, as imDex are stable and can be easily stored in vitro, they may be a good substitute for imDCs in inducing immune tolerance. However, there are few reports on the utility of imDex as an immunosuppressant, and only two findings show that treatment with imDex alone induces limited immunosuppressant activity without inducing tolerance [13], [14].

In this study, we analyzed the effect of allogenetic donor imDex, purified from mouse bone marrow (BM), on tolerance induction in a mouse model of heart transplantation. We found that low doses (10 μg) of donor imDex could significantly prolong cardiac allograft survival, but this was limited. To increase graft survival, therefore, we used imDex together with a subtherapeutic regimen (1 mg/kg/d) of rapamycin. Impressively, the combination of imDex and rapamycin led to donor specific cardiac allograft tolerance, and this effect was accompanied by decreased anti-donor antigen cellular response and an increased percentage of spleen CD4^+^CD25^+^ T cells in recipients. These findings demonstrate that donor imDex in combination with an immunosuppressant can induce donor specific allograft tolerance and thus prolong cardiac allograft survival.

## Results

### Characterization of Exosomes Derived from DCs

**Figure 1:**
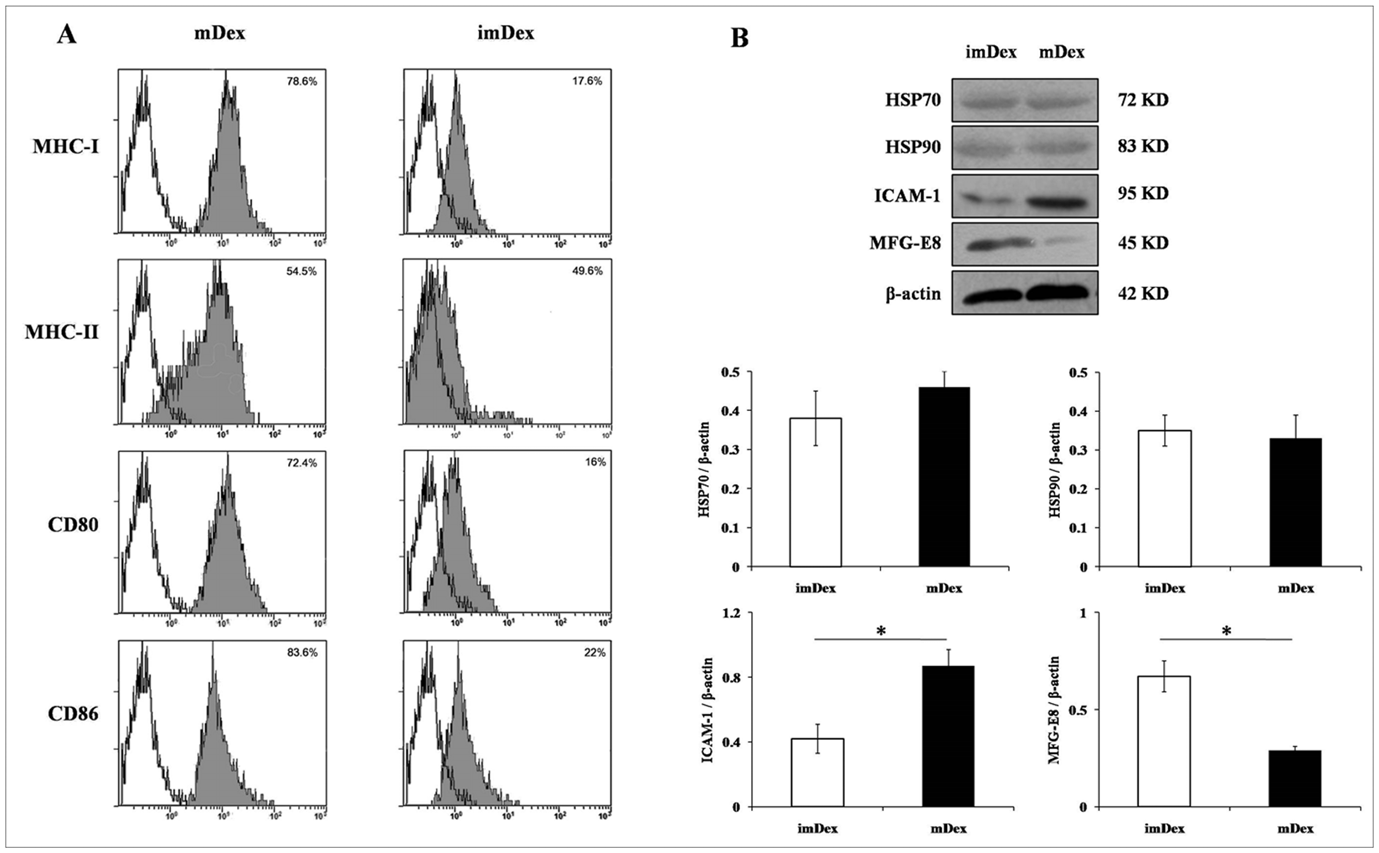
Characterization of exosomes derived from DCs. (A) The phenotypic profile of imDex and mDex were analyzed by cytofluorometry. The two kinds of the exosomes were coated on surfactant-free white aldehyde sulfate latex beads, then stained with FITC-coupled monoclonal antibodies against MHC class I, II and CD80, CD86. The isotype monoclonal antibody of IgG was used as control. The results are representative of four independent experiments. (B) Western blotting analysis of imDex and mDex using monoclonal antibodies specific for HSP70, HSP90, ICAM-1, and MFG-E8. The signal intensities in the blot were assessed by densitometry and are represented in the graphs. One representation of three independent experiments is shown. * *P*<0.01.

Eight days after culturing in the presence of granulocyte macrophage colony-stimulating factor (GM-CSF) and recombinant interleukin-4 (IL-4), murine BM cells developed a typical DC profile as reported by Lutz *et al*. [16]. Phenotypic profiles of mature DCs (lipopolysaccharide (LPS)-treated) and imDCs (untreated) analyzed by fluorescence-activated cells sorting (FACS). Mature DCs (mDCs) expressed high levels of MHC class I, class II, and co-stimulatory CD80 and CD86 compared with imDCs. The DC marker NLDC-145 showed exclusive staining in mDCs but was reduced in imDCs, whereas CD11c was moderately expressed in both mDCs and imDCs. These data proved that DCs were immature throughout the culture period in the presence of GM-CSF and IL-4. Exosomes were purified from culture supernatants of mDCs and imDCs by ultrafiltration and ultracentrifugation [17]. We obtained about 0.35±0.1 μg and 0.94±0.2 μg of exosome proteins (as measured by Bradford assay) from one million mDCs and imDCs, respectively. The morphology of exosomes observed by electron microscopy. The purified exosomes showed typical heterogeneous vesicle structures ranging from 60 to 90 nm in diameter. A typical phenotypic profile of imDex and mDex is shown in Figure 1A. Lower levels of MHC class I and co-stimulatory CD80 and CD86 were expressed in imDex than mDex. MHC class II, which was highly expressed in mDex, was slightly down-modulated in imDex. Additionally, we analyzed the expression of HSP70, HSP90, MFG-E8, and ICAM-1 by western blotting (Figure 1B). HSP70 and HSP90 were present in similar amounts in imDex and mDex, but ICAM-1 expression was relatively higher and MFG-E8 was relatively lower in mDex.

### Administration of Donor imDex in Combination with Low-Dose Rapamycin Prolongs Survival of Cardiac Allografts

**Figure 2:**
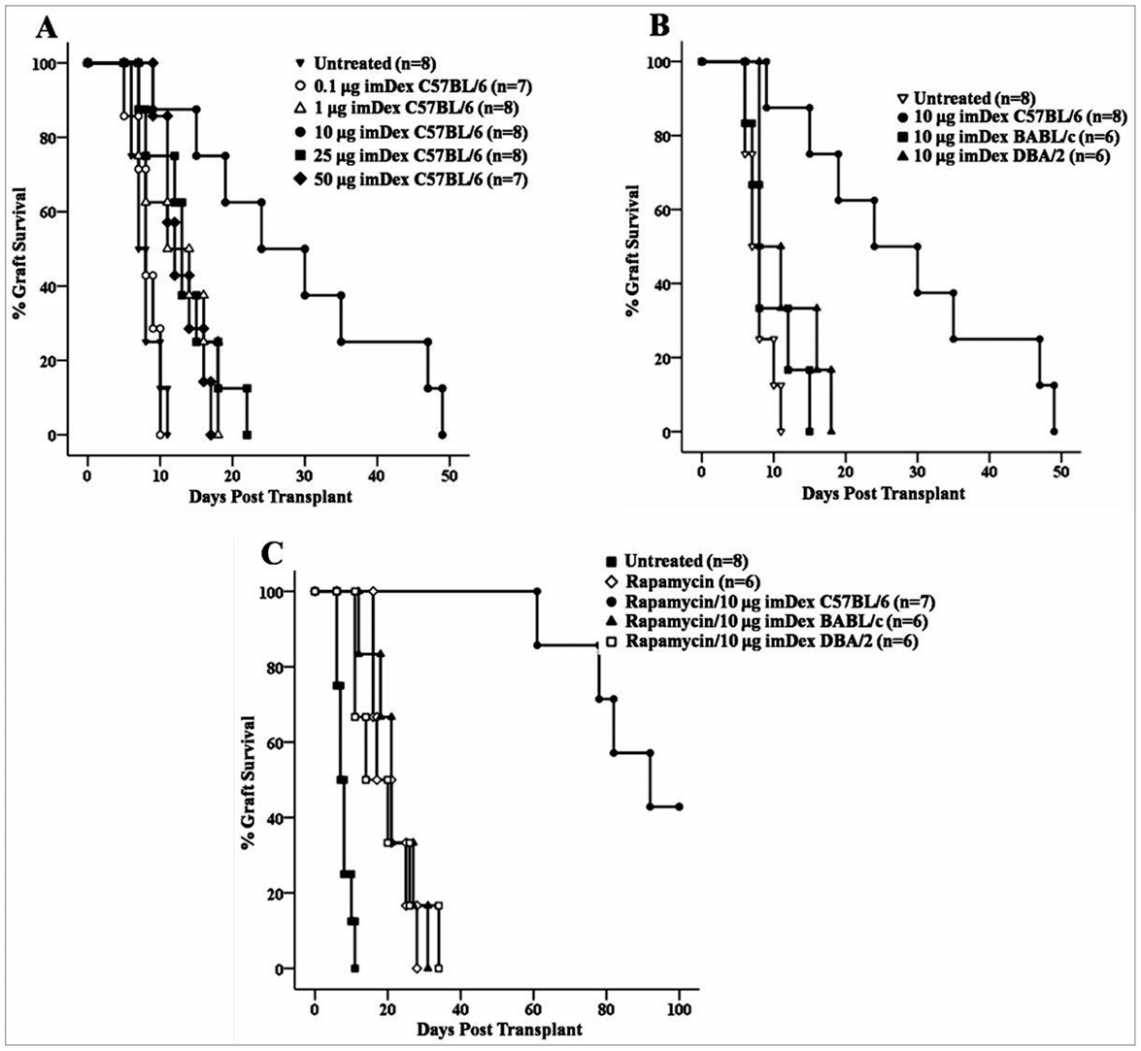
Administration of low-dose donor imDex with a subtherapeutic rapamycin promotes cardiac allograft survival. Different imDex concentrations were injected via caudal vein 7 d before, the day of, and 7 d after transplantation, and 1 mg/kg/d rapamycin was injected via caudal vein for 11 consecutive days (days –3 to 7) with or without imDex. (A) The cardiac allograft survival for different donor imDex concentrations in the absence of rapamycin. (B) ImDex (10 μg) of the donor (C57BL/6), recipient (BALB/c), or third-party (CBA/H) were injected into recipient mice. (C) The allograft survival following rapamycin treatment alone or with 10 μg donor, recipient, or third-party imDex.

Some reports have shown that administration of donor imDex before transplantation significantly prolongs allograft survival of heart or intestine in rat transplantation models [13], [14]. However, transplant immunological tolerance or long-term graft survival was not achieved, and the role of imDex treatment in allograft transplantation was not completely clear. Thus, we treated recipients with donor imDex alone or in combination with low-dose rapamycin and analyzed the effects on survival of cardiac allografts. First, we tested the effects of different doses of donor imDex. We established a murine model of cardiac allotransplantation, and treated BALB/c recipients with 0.1, 1, 10, 25, or 50 μg imDex derived from a C57BL/6 donor. The imDex were injected via caudal vein 7 d before, the day of, and 7 d after transplantation. As shown in Table 1 and Figure 2A, treatment with 0.1 or 1 μg of donor imDex (median survival time (MST): 8 d for 0.1 μg, n = 7; 12.5 d for 1 μg, n = 8) did not prolong allograft survival compared with the untreated recipients (MST: 7.5 d, n = 8); treatment with 10 μg imDex (MST: 27 days, n = 8), however, significantly prolonged allograft survival (*P*<0.001). Notably, allograft survival decreased when the dose of donor imDex increased above 10 μg. We found that treatment with 25 μg imDex (MST: 14 d, n = 8) also prolonged allograft survival compared with untreated recipients (*P* = 0.042), but the effect was significantly weaker than for treatment with 10 μg imDex (*P* = 0.001). Finally, treatment with 50 μg imDex (MST: 12 d, n = 7) did not affect allograft survival compared with the untreated recipients (*P* = 0.192). Thus, treatment of the recipient mice with a low-dose (10 μg) of donor imDex before and after transplantation inhibited the immunological rejection of the cardiac allograft and significantly prolonged allograft survival.

**Table 1:** Cardiac allograft survival after treatment with imDex or imDex+rapamycin.

Treatment	Median (days)	Graft survival days	*P* value
Untreated*	7.5	6×2, 7×2, 8×2, 10, 11	
0.1 μg imDex C57BL/6	8	5, 7, 8×2, 9, 10×2,	>0.05^a^
1 μg imDex C57BL/6	12.5	7×2, 8, 11, 14, 16, 18×2	>0.05^a^
10 μg imDex C57BL/6	27	9, 15, 19, 24, 30, 35, 47, 49	<0.001^a^
25 μg imDex C57BL/6	14	7, 8, 12, 13, 15, 18, 22, 29	<0.05^a^, <0.01^b^
50 μg imDex C57BL/6	12	9, 11×2, 12, 14, 16, 17	>0.05^a^
10 μg imDex BALB/c	8	6, 7, 8×2, 12, 15,	>0.05^a^, <0.001^b^
10 μg imDex CBA/H	9.5	8×3, 11, 16, 18	>0.05^a^, <0.01^b^
Rapamycin	19	16×2, 17, 21, 25, 28	<0.05^a^, <0.001^c^
Rapamycin/10 μg imDex C57BL/6	92	61, 78, 82, 92, >100×3	<0.001^a^
Rapamycin/10 μg imDex BALB/c	21	12, 18, 21×2, 27, 31	<0.05^a^, <0.001^c^
Rapamycin/10 μg imDex CBA/H	17	11×2, 14, 20, 26, 34	<0.05^a^, <0.001^c^

BALB/c recipients were transplanted with a C57BL/6 heart. Recipients were treated with imDex or imDex/rapamycin.

* Treated with physiological saline.

*^a^*Compared with untreated mice.

*^b^*Compared with 10 μg imDex C57BL/6-treated mice.

*^c^*Compared with 10 μg imDex C57BL/6/rapamycin-treated mice.

To determine whether this effect was donor specific, we injected 10 μg imDex derived from BALB/c (recipient) or CBA/H (third-party) mice into BALB/c recipient mice, but we found that neither prolonged cardiac allograft survival (MST: 8 d for BALB/c imDex and 9.5 d for CBA/H imDex; Figure 2B and Table 1), indicating that the effect was donor specific. Notably, increased doses of donor imDex did not induce accelerated rejection despite the negative effect on allograft survival, suggesting that administration of donor imDex via caudal vein before transplantation does not sensitize the recipients.

Although a low dose (10 μg) donor imDex significantly prolonged cardiac allograft survival in our experiments, this effect was limited and merely yielded MST of 27 d and a maximum survival of 51 d. To improve allograft survival, we used 10 μg donor imDex together with a short course of immunosuppression intended to develop tolerance in the immunological microenvironment in vivo. Rapamycin–the mammalian target of rapamycin (mTOR) inhibitor -blocks DC maturation [18], [19], inhibits capacity of DC to stimulate T cells even following exposure to LPS [20], and prolongs organ allograft survival [21]. Thus, we hypothesized that 10 μg donor imDex combined with a short course of minimally effective rapamycin could induce immunological tolerance and effectively promote allograft survival. To test this hypothesis, we treated recipient mice with a subtherapeutic regimen of 1 mg/kg/d rapamycin injected via caudal vein for 11 consecutive days (days -3 to 7) with or without donor imDex. We found that this combination dramatically induced long-term cardiac allograft survival (MST: 92 d, n = 7; Table 1 and Figure 2C), whereas the subtherapeutic regimen of rapamycin alone did not significantly prolong allograft survival (MST: 19 d, n = 6; Table 1 and Figure 2C). We also confirmed that this effect was specific for donor antigen by combining injection of the subtherapeutic regimen of rapamycin with 10 μg imDex derived from either recipient or third-party mice and found that these treatments had no significant effect on allograft survival (MST: 21 d for imDex/rapamycin-cotreatment, n = 6; 17 d for third-party imDex/rapamycin-cotreatment, n = 6; Figure 2C and Table 1).

### The Combination of Rapamycin and Donor imDex Decreases the Cellular Response against Donor Antigen Post-Transplantation

**Figure 3:**
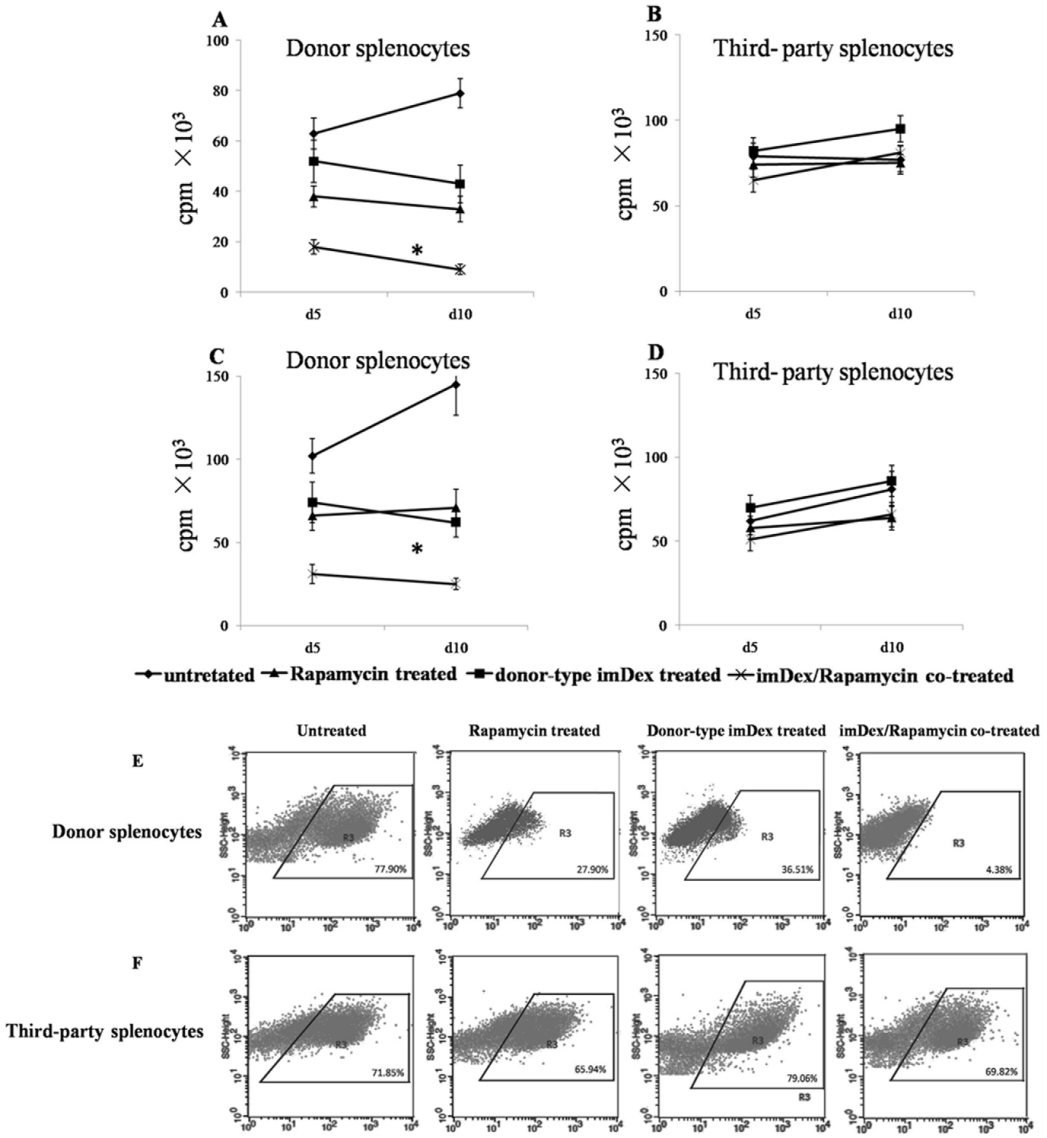
Donor imDex combined with rapamycin inhibits proliferation and activation of alloreactive T lymphocytes against donor antigen. At 5 or 10 d post-transplantation, 5×10^4^ total splenocytes (A-B) or purified T lymphocytes (C-D) from untreated, donor imDex-treated, rapamycin-treated or imDex/rapamycin-cotreated recipients (responder cells) were incubated with 2×10^4^ donor or third-party splenocytes (stimulator cells) for 72 h. Label (1 μCi [^3^H]-thymidine) was added for the last 18 h of culture, and incorporation was determined using a liquid scintillation counter. Upregulation of CD69 on the four groups of responder cells (10 d after transplantation) was measured by FACS 24 h after simulator cell addition (E-F). One representation of four independent experiments is shown. * *P*<0.01.

To determine the mechanism of the cardiac allograft tolerance induced by imDex/rapamycin-cotreatment, we assessed the anti-donor cellular response in recipients after transplantation; at 5 or 10 d post-transplantation, total splenocytes and purified splenic T lymphocytes were separated from different recipients, which included untreated recipients (n = 6, 3 mice each for d5 and d10), recipients of donor imDex (n = 5, 3 mice for d5 and 2 mice for d10) or rapamycin (n = 6, 3 mice each for d5 and d10) alone, and imDex/rapamycin-cotreated recipients (n = 6, 3 mice each for d5 and d10). These splenocytes and splenic T lymphocytes were responder cells. Stimulator cells were splenocytes from donor (C57BL/6) or third party (CBA/H) treated with mitomycin C for 24 hours (h). Responder cells (5×10^4^) were co-cultured with stimulator cells (2×10^4^) for 72 h. Cell proliferation was quantified by incubating the cells with 1 μCi [^3^H]-thymidine during the last 18 h of co-culture. Total splenocytes from imDex/rapamycin-cotreated recipients induced a significant decrease in proliferation against donor splenocytes compared with responder cells from untreated recipients (d5: *P*<0.001, d10: *P*<0.001). Total splenocytes from recipients treated with donor imDex or rapamycin alone also negatively affected the anti-donor antigen cellular response compared with splenocytes from untreated recipients (d5: *P* = 0.164, d10: *P* = 0.008, for donor imDex; d5: *P* = 0.004, d10: *P*<0.001, for rapamycin), but splenocytes from recipients treated with donor imDex or rapamycin alone were 4- to 5-fold less efficient than those from recipients co-treated with imDex and rapamycin (Figure 3A). However, splenocytes from the four groups proliferated equivalently against the third-party CBA/H splenocytes (Figure 3B). These results were also observed in proliferation of purified T lymphocytes against donor or third-party splenocytes (Figure 3C–D). Furthermore, we assessed activation of T lymphocytes by measuring upregulation of the early activation marker CD69 after splenic T lymphocytes purified from different recipients (10 d after transplantation) were co-cultured with stimulator cells for 24 h. T lymphocytes of imDex/rapamycin-cotreated recipients expressed lower levels of CD69 compared with those of untreated recipients (Figure 3E, *P*<0.001). The levels of CD69 were also downregulated on T lymphocytes of donor imDex-treated or rapamycin-treated recipients compared with those of untreated recipients, but were significantly different than those of imDex/rapamycin-cotreated recipients (*P* = 0.013 for rapamycin; *P = *0.025 for imDex). However, CD69 was expressed similarly on splenocytes of the four groups against third-party CBA/H lymphocytes (Figure 3F). We concluded that activation and proliferation of T lymphocytes against donor antigen are more efficiently inhibited by rapamycin/imDex-cotreatment than by donor imDex treatment alone.

### Treatment with Donor imDex and Rapamycin Induces Spleen CD4^+^CD25^+^ T Cells in Recipient Mice

**Figure 4:**
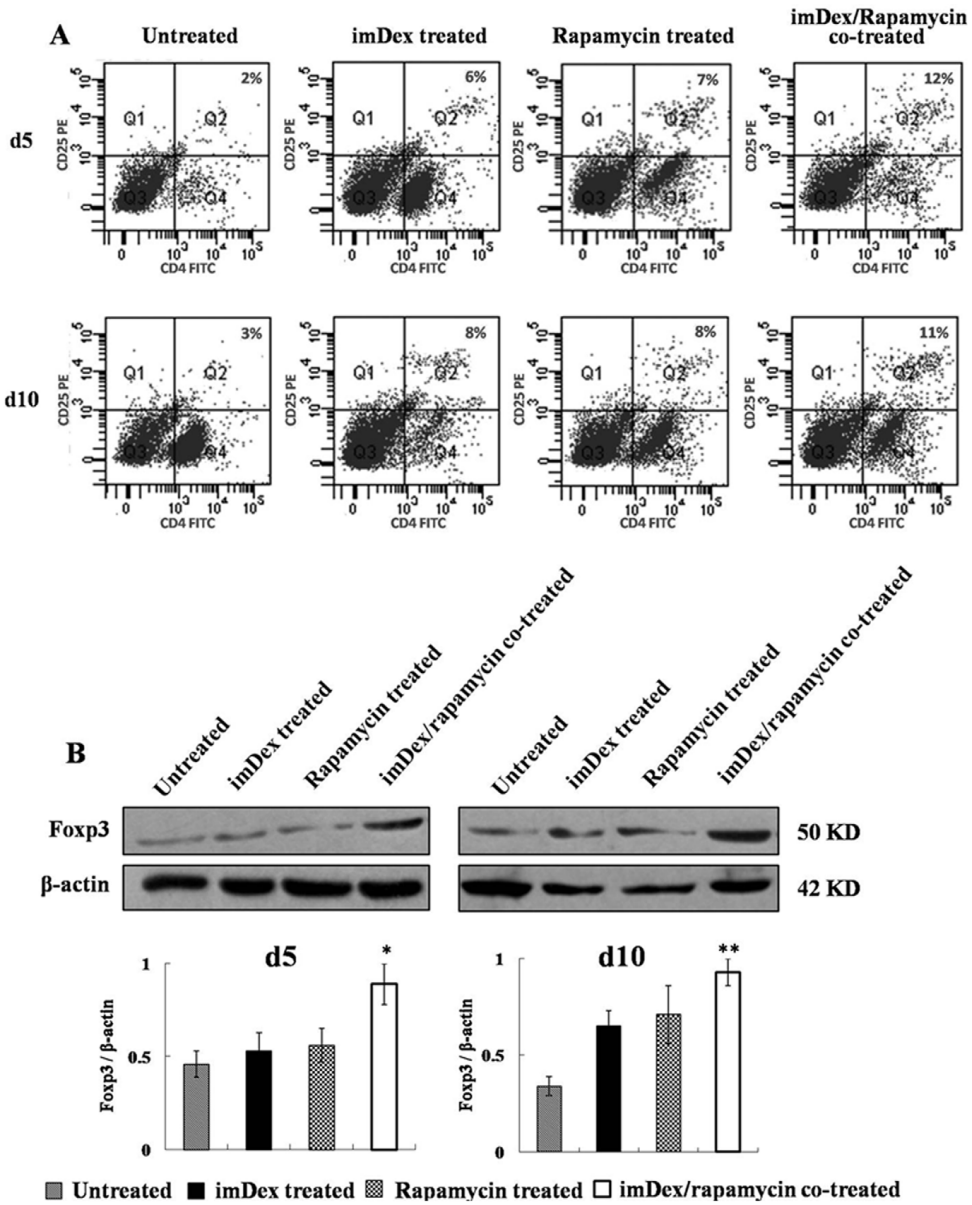
FACS and western blotting analysis of splenic CD4^+^CD25^+^Foxp3^+^ T cells. Splenic T lymphocytes were isolated from untreated recipients (n = 6), recipients treated with donor imDex (n = 6) or rapamycin (n = 6) alone, and imDex/rapamycin-cotreated (n = 6) recipients at d5 or d10 after transplantation. (A) The percentages of splenic CD4^+^CD25^+^ T cells from the four groups of recipient mice were calculated from flow cytometric analysis. The number in the upper-right quadrant of each histogram indicates the percentage of CD4^+^CD25^+^ T cells. Results were obtained from three mice per condition, and one representative of four independent experiments is shown. (B) Foxp3 versus β-actin western blotting analysis of splenic T lymphocytes from the groups of recipients upper. Densitometry shows fold induction of Foxp3:β-actin ratio. Bars  =  mean ± SD (n = 3), * *P*<0.01, ** *P*<0.001. One representation of three independent experiments is shown.

The inhibition of T lymphocyte activation and proliferation against donor antigen suggested that treatment with imDex plus rapamycin may favor expansion of the percentage of CD4^+^CD25^+^Fox3^+^ regulatory T (Treg) cells. To this end, purified splenic T lymphocytes were collected from untreated recipients (n = 6), recipients of donor imDex (n = 6) or rapamycin (n = 6) treatment alone, and imDex/rapamycin-cotreated recipients (n = 6) 5 or 10 d after transplantation. The percent of CD4^+^CD25^+^ T cells was analyzed by FACS, and the Foxp3 expression level was analyzed by western blotting. As shown in Figure 4A, treatment with imDex/rapamycin resulted in a notable increase in the proportion of CD4^+^CD25^+^ T cells compared with that of the untreated group (d5: 12% ±0.9% versus 2% ±0.4%, *P*<0.001; d10: 11% ±1.4% versus 3% ±0.2%, *P*<0.001). Treatment with donor imDex or rapamycin alone also increased the proportion of CD4^+^CD25^+^ T cells, but neither treatment was as efficient as imDex/rapamycin-cotreatment (d5: *P*<0.001, d10: *P*<0.001, for donor imDex; d5: *P* = 0.001, d10: *P*<0.001, for rapamycin). Western blotting showed enhanced production of Foxp3 in purified splenic T lymphocytes from imDex/rapamycin-cotreated recipients as compared with untreated recipients (d5: *P* = 0.007, d10: *P*<0.001; Figure 4B). Moreover, we found a striking 1.7- to 1.9-fold decrease in Foxp3 expression in recipients treated with donor imDex or rapamycin alone as compared with recipients cotreated with imDex and rapamycin. These data indicated that the combination of imDex and rapamycin may inhibit proliferation and activation of alloreactive T lymphocytes, which is closely related to increased proportion of the spleen CD4^+^CD25^+^ T cells in recipients.

### Administration of Donor imDex in Combination with Low-Dose Rapamycin Prevents Acute Cardiac Allograft Rejection Lesions

**Figure 5:**
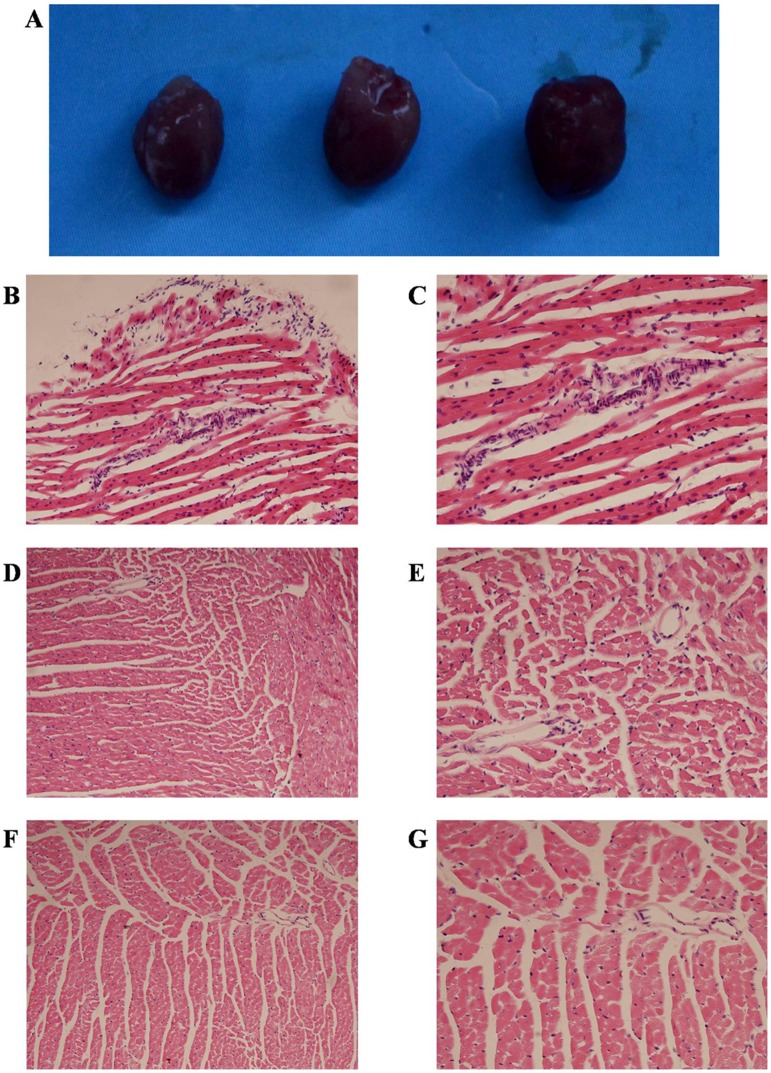
Signs of acute rejection in cardiac allografts. Cardiac allografts of untreated and imDex/rapamycin-cotreated recipients were harvested at 7 d post-transplantation, and transplantation of a syngenetic heart (BALB/c to BALB/c) was used as control. (A) The left heart is from the syngenetic transplantation recipient, the middle heart is from the imDex/rapamycin-cotreated recipient, and the right heart is from the untreated recipient. Paraffin sections were stained with hematoxylin-eosin and observed by optical microscope. Representative allografts of untreated recipients (B and C), allografts of imDex/rapamycin-cotreated recipients (D and E), and control specimens from syngenetic transplantation recipients (F and G) are shown.

To determine the degree of the benefit of the combination of donor imDex with rapamycin for preventing acute cardiac allograft rejection lesions, we harvested hearts of untreated (n = 3) and imDex/rapamycin-cotreated (n = 3) recipients 7 d post-transplantation and examined the histology of these allografts. A heart of syngenetic transplantation (BALB/c to BALB/c) was used as a control. Upon gross examination, the heart of the untreated recipient was obviously congestive and edematous, but the imDex/rapamycin-cotreated recipient’s heart was similar to the control (Figure 5A). As compared with the control (Figure 5F and 5G), microscopic examination of allografts of the untreated group showed severe lymphocyte infiltration and interstitial edema, which are signs of acute graft rejection (Figure 5B and 5C). By contrast, the allografts of imDex/rapamycin-cotreated recipients displayed minor pathological changes of acute graft rejection (Figure 5D and 5E). These results indicated that administration of donor imDex in combination with rapamycin can efficiently prevent acute cardiac allograft rejection.

### Tolerance Induced by Cotreatment with imDex and Rapamycin Is Donor Specific and Transferable

To assess whether the recipients cotreated with imDex and rapamycin displayed donor specific tolerance, these mice had a second heart from donor mice (C57BL/6, n = 3) or third-party mice (CBA/H, n = 3) transplanted in the neck. Three naïve BALB/c mice that received hearts from C57BL/6 mice were used as controls. As shown in Table 2, the cardiac allografts from donor mice survived longer than the hearts from third-party mice and control mice (MST: >100 d for donor hearts; 21 d for third-party hearts; 9 d for control group).

**Table 2:** Survival of the second cardiac allograft for tolerant imDex/rapamycin-cotreated recipients.

Recipients	Second graft	Median (days)	Graft survival days	*P* value
Naïve BALB/c	C57BL/6	9	7, 9, 12	
Tolerant imDex/rapamycin- cotreated BALB/c	C57BL/6	>100	75, >100×2	<0.001a, b
Tolerant imDex/rapamycin- cotreated BALB/c	CBA/H	21	11, 21, 24	>0.05^a^

Donor imDex/rapamycin-cotreated recipients were transplanted with a secondary C57BL/6 or CBA/H heart in the neck. Naïve BALB/c recipients that received C57BL/6 hearts were used as controls.

*^a^*Compared with naïve BALB/c group.

*^b^*Compared with cardiac allograft of CBA/H.

To determine whether the tolerance in imDex/rapamycin-cotreated recipients was transferable, we injected serum or splenocytes from tolerant recipients (n = 3) or naïve mice (n = 3) into naïve irradiated BALB/c mice via the caudal vein and then transplanted C57BL/6 mouse hearts into them. As shown in Table 3, the administration of splenocytes derived from tolerant mice significantly prolonged the cardiac allograft survival (MST: 83 d), but the splenocytes derived from naïve BALB/c mice did not exhibit this effect (MST: 16 d). We also found that the transfer of serum derived from the tolerant recipients prolonged the allograft survival to a limited extent compared with the splenocytes of tolerant recipients (MST: 44 d). These data suggested that tolerance induced by imDex/rapamycin-cotreatment is donor specific and transferable, and it may be mediated by recipient immunoregulatory cells but not by recipient serum.

**Table 3:** Cardiac allograft survival for BALB/c recipients injected with splenocytes or serum from tolerant imDex/rapamycin-cotreated recipients.

Treatment	Median (days)	Graft survival days	*P* value
Untreated*	15	12, 15, 21	
Splenocytes from naïve BALB/c	16	15, 16, 19	>0.05 a
Splenocytes from imDex/rapamycin- cotreated recipients	83	83, 88, >100	<0.001 a, b
Serum of naïve BALB/c	19	14, 19, 20	>0.05 a
Serum of imDex/rapamycin-cotreated recipients	44	38, 44, 57	<0.001 a

All naïve BALB/c mice were irradiated with 3 Gy before transfer of 5×10^7^ splenocytes or 0.8 mL serum.

* Treated with physiological saline.

*^a^*Compared with untreated group.

*^b^*Compared with serum of imDex/rapamycin-cotreated recipients.

## Discussion

The principle method for generating immature BM-DCs with GM-CSF plus IL-4 was first reported by Inaba *et al*. [22] and Sallusto *et al*. [23]. So, we applied this conventional method to obtain imDCs that expressed lower MHC class I and co-stimulatory CD80 and CD86 than DCs stimulated with LPS, and this is consistent with many reported data [16], [24]. Next, we extracted exosomes from the culture supernatant of imDCs or mDCs and comparatively analyzed the differences in morphology and protein composition between the two kinds of Dex. Although there were no significant differences in morphology, there was a large difference in protein composition. Similar to imDCs, imDex expressed the “tolerance” phenotype with low levels of MHC class I, CD80, and CD86 and moderate levels of MHC class II molecules. Meanwhile, imDex expressed lower levels of ICAM-1 than mDex, which is necessary for the immune activity of mDex and as a major exosomal protein for inducing immune rejection to skin allografts [9]. These results suggest that imDex have the potential to induce immune tolerance. Thus, we injected donor imDex into recipients before and after transplantation, and found that treatment with 10 μg of donor imDex significantly prolonged cardiac allograft survival, but this effect decreased when imDex dose was increased. This effect was donor specific because imDex from syngenetic mice or third-party mice had no such effect.

The above results indicate that, compared with imDCs, use of imDex to induce immune tolerance has several advantages. First, as a source of donor antigens, imDex can be frozen long-term in vitro without affecting biological activity [25], so imDex can be prepared quickly and used immediately when necessary. Second, because only a small amount of imDex is needed to prolong allograft survival, it can be used repeatedly on recipients if necessary. Third, imDex do not undergo maturation in vivo, which is a crucial drawback in current DC-based therapies for tolerance induction. Finally, large doses of donor imDex (50 μg) do not accelerate immune rejection, so it is safe to treat recipients with donor imDex through the veins before transplantation, and the donor imDex do not sensitize recipients against donor antigens.

Previous reports showed that the role of exosomes in developing tolerance or immunity depends not only on the stage and type of the secreting cells but also on the state of the immune microenvironment where the exosome-APC interaction takes place [11], [26]. In our study, although a low dose of donor imDex significantly prolonged cardiac allograft survival, this effect was limited and was insufficient to induce immune tolerance. We considered that donor imDex should be combined with non-specific immunosuppressive therapy to develop a tolerance microenvironment in vivo. Rapamycin was thus used to favor the presentation of alloantigens in a tolerogenic fashion because it blocks T-cell activation, inhibits DCs maturation, and selectively allows for proliferation while fostering the suppressive function of Foxp3^+^ Treg cells [27], [28]. We observed that the combination of low-dose rapamycin and donor imDex induced long-term cardiac allograft survival, but rapamycin alone did not have this effect. This result suggests that, in a steady and tolerogenic state, imDex-delivered donor antigens will prevent immune rejection and promote immune tolerance.

Although the exact mechanism remains unclear, there is increasing evidence that donor APC-derived exosomes administered intravenously might participate in induction and maintenance of peripheral T-cell tolerance. Morelli *et al*. [29] reported that circulating allogeneic exosomes are efficiently captured by recipient splenic DCs without triggering their activation, and they deliver donor MHC molecules to host T cells, which induce donor specific T-cell tolerance in recipients. In this study, we found that total splenocytes and splenic T cells from imDex/rapamycin-cotreated recipients showed a significant decrease in proliferation and activation response against donor splenocytes. These data suggest that combined treatment with donor imDex and rapamycin inhibits T-cell activation and may induce anergy of alloreactive T cells for donor antigen.

We also observed that the combination of imDex and rapamycin notably increased the percentage of CD4^+^CD25^+^ T cells and up-regulated Foxp3 expression in recipient splenic T cells. Furthermore, we found that the tolerance in imDex/rapamycin-cotreated recipients could be transferred to naïve syngenetic mice by injecting the recipients’ splenocytes but not serum. These results suggest that the tolerance may be mediated by immunoregulatory cells of recipients. But on the other hand, we did not confirm whether these CD4^+^CD25^+^ T cells are Treg cells or not. We didn’t analyze their alloantigen-specificity and regulatory functions experimentally either. Because of this, we cannot confirm the immune tolerance we induced are directly related to the CD4^+^CD25^+^Foxp3^+^ Treg cells. However, only a few studies have focused on the effect of exosomes on Treg cells. Wang *et al*. reported that exosomes derived from thymus can induce Treg cell proliferation in vivo [30]. Cai *et al*. described utilizing exosomes that were derived from TGF-β1 gene-modified BM-DCs to induce Foxp3^+^ Treg cells and decrease the proportion of Th17 cells in a mouse model of inflammatory bowel disease [31]. In the future, we will do more study on the number, phenotype and function of splenic CD4^+^CD25^+^ T cells in the tolerant recipients to find the relation between these cells and Treg cells.

Taken together, our study demonstrates that the combination of donor imDex and low-dose rapamycin treatment can induce donor specific immune tolerance in a murine heterotopic cardiac transplantation model and prolong allograft survival. The tolerance can persist long term and be transferred to naïve syngenetic recipients by injecting tolerific immunocytes. Our study reports a novel therapeutic approach for inducing transplant immune tolerance, but mechanisms such as inter-regulation among imDex, DCs, and Treg cells should be examined in future studies.

## Materials and Methods

### Ethnics Statements

All animals experiments were performed in accordance with a guideline from the Administration of Animal Experiments for Medical Research Purposes issued by the Ministry of Health of China. The protocol was approved by the Animal Experiment Administration Committee of Fourth Military Medical University. All surgery was performed under sodium pentobarbital anesthesia and accomplished in a clean surgery room with sterilized instruments. All efforts were made to minimize the suffering of the mice during the experiments.

### Mice

Six- to twelve- week-old male C57BL/6 (H-2b), male BALB/c (H-2d) and male CBA/H (H-2k) mice were obtained from the Experimental Animal Center of The Fourth Military Medical University and housed in specific pathogen-free conditions.

### Antibodies

The monoclonal antibodies used for cytofluorometric analysis were fluorescein isothiocyanate (FITC)-conjugated anti-MHC class I, anti-MHC class II, anti-CD80, anti-CD86, anti-CD4, anti-CD69 were purchased from Bioscience (San Diego, CA, USA), and FITC-conjugated anti-CD11c, FITC-conjugated anti-NLDC-145, and phycoerythrin (PE)-conjugated anti-CD25 were purchased from Abcam (Cambridge, UK). The corresponding isotype antibodies were control. Western blotting utilized monoclonal antibodies (all from Santa Cruz Biotechnology, Santa Cruz, CA) against HSP70, HSP90, ICAM-1, MFG-E8, and Foxp3; horseradish peroxidase (HRP)-conjugated secondary antibodies (Santa Cruz Biotechnology) were used.

### Cultivation of BMDCs in vitro

DCs were generated from BM progenitor cells as described by Muthana *et al*. [32]. In brief, BM cells were collected from femurs and tibias of mice. To remove the adherent macrophages, the BM cells then were cultured for 24 h in complete RPMI 1640 medium (Invitrogen, Carlsbad, CA, USA) supplemented with 10% endotoxin-free fetal bovine serum (Gibco, Carlsbad, CA, USA) that had been centrifuged (110,000×g overnight) to deplete contaminating vesicles and protein aggregates. The non-adherent cells were culture for 8 d in fresh complete medium containing recombinant mouse GM-CSF and IL-4 (10 ng/mL, Peprotech, Rocky Hill, NJ). The cells and supernatant of culture medium were harvested. Then, part of the harvested cells were re-cultured in fresh complete medium containing LPS (10 μg/mL, Sigma, St. Louis, M.O.) for 48 h, and the cells and supernatant were also harvested.

### Exosomes Preparation

Exosomes were purified from the culture supernatant of mDCs and imDCs by ultrafiltration and ultracentrifugation as described by Lamparski *et al*. [17]. In brief, the supernatants were first centrifuged at 300×g for 10 min and then at 1,200×g for 30 min, each at 4°C. The clarified supernatant was concentrated by centrifugation for 30 min at 4,000×g, at 4°C, in a pre-rinsed Centricon Plus-80 capsule filter (MW 100 KDa, Millipore, Danvers, MA). The ultrafiltrate was underlayed with 30% sucrose/D_2_O density cushion, followed by ultracentrifugation at 110,000×g and 4°C for 1 h. The cushion was collected and diluted in Dulbecco’s phosphate-buffered saline (DPBS), and then concentrated by centrifugation for 30 min at 4,000×g at 4°C in a pre-rinsed 100 KDa MWCO Millipore Centricon Plus-80 capsule filter. The protein amounts were quantified by Bradford assay (Bio-Rad, Hercules, CA, USA).

### Electron Microscopy

The sample of exosomes was loaded onto a copper grid at room temperature. After the exosomes precipitated for 3 min, the sample liquid was sucked up slowly with filter paper from the side of the copper mesh. The sample was then counterstainded with 2% phosphotungstic acid solution (30 μL, pH 6.8) for 10 min at room temperature. The sample liquid was sucked up again, and the sample was placed under an incandescent lamp for 5 min. Finally, the exosomes sample was examined with a CM20 Twin Philips electron microscope (Phillips Electronic Instruments, Mahway, NJ).

### Flow Cytometry

The two kinds of DCs were harvested, washed, and resuspended in PBS, 1% bovine serum albumin, 5% heat-inactivated normal rabbit serum, and 0.1% sodium azide. Cells were incubated for 45 min at 4°C with FITC-conjugated anti-MHC class I, anti-MHC class II, anti-CD80, anti-CD86, anti-CD11c, or anti-NLDC-145. The two kinds of exosomes were incubated with aldehyde/sulfate latex beads (Interfacial Dynamics, Portland, OR, USA) at 4°C overnight. The reaction was stopped with 100 mM glycine for 30 min. The beads were washed three times in flow buffer (3% fetal bovine serum in PBS) and stained with FITC-conjugated anti-MHC class I, anti-MHC class II, anti-CD80, or anti-CD86. The beads and cells were analyzed by flow cytometry using a FACSCalibur (BD Immunocytometry Systems, Franklin Lakes, NJ, USA).

### Western Blotting

Proteins (10 μg) from the two kinds of exosomes were separated by 10% SDS-PAGE, transferred onto PVDF membranes (Millipore, USA). After blocking with 5% nonfat dry milk in PBS, the membrane was incubated with anti-HSP70, anti-HSP90, anti-ICAM-1, and anti-MFG-E8 followed by HRP-conjugated secondary antibodies and detected by enhanced chemiluminescence using X-ray films.

### Transplantation

Celiac heart transplants were performed using a modified technique as described by Niimi [33]. C57BL/6 mice served as heart donors and BALB/c mice as allograft recipients. After the donor mice were anesthetized, the inferior vena cava, azygos vein, superior vena cava, ascending artery and pulmonary artery were ligated with 7-0 silk sutures and cut in order. An end-to-side anastomosis was performed with 10-0 surgical sutures between the donor ascending artery with the recipient abdominal artery and between the donor pulmonary artery with the recipient inferior vena cava. Cervical heterotopic heart transplants were performed according to the Chen technique [34]. The donor ascending artery was sutured end-to-side to the recipient right common carotid artery, and the donor pulmonary artery was anastomosed to the recipient right external jugular vein. Cardiac graft function was tested daily by transabdominal palpation. Rejection was defined as a complete cessation of palpable beating. Cardiac allografts that stopped beating within 2 d were regarded as technical failures and excluded from further analysis.

### In vitro T-Cell Stimulation Assay

Five or ten days after transplantation, total splenocytes of recipient BABL/c mice were sterilely extracted, and T cells were isolated from the splenocytes using nylon wool columns (Fenwall Laboratories, Lake Zurich, IL, USA); the two kind of cells were responder cells. Responder cells (5×10^4^) were seeded into 96-well round-bottom plates (Costar, Santa Fe Springs, CA, USA) with stimulatory cells that were donor (C57BL/6) or third-party (CBA/H) splenocytes treated with mitomycin C 24 h. [^3^H]-thymidine (1 μCi/well) was added 72 h later for 18 h, and [^3^H]-thymidine incorporation was countered by liquid scintillation. Additionally, the T cells were harvested after co-cultured with stimulatory cells and stained with anti-CD69. T cells were then analyzed by flow cytometry.

### Spleen CD4^+^CD25^+^ T Cells and Foxp3 Protein Quantification

Five or ten days after transplantation, splenic T cells were purified from recipient mice and stained with FITC-conjugated anti-CD4 and PE-conjugated anti-CD25, and then analyzed by flow cytometry. Foxp3 protein of splenic T cells was measured by western blotting.

### Assessment of Histological and Morphometric Changes

Seven days after transplantation, specimens of cardiac allografts from different recipients were obtained and fixed with paraformaldehyde and then embedded in paraffin. Sections (5 μm) were stained with hematoxylin-eosin to assess morphological changes. Transplantation of a syngenetic heart (BALB/c to BALB/c) was used as a control.

### Statistics Analysis

One-way analysis of variance was used for analysis within groups. The Kaplan and Meier method was used to calculate the survival curves, and the log-rank test was used to compare animal survival in different groups of mice using SPSS for Windows 17.0 software. *P*<0.05 was considered significant.

## References

[1] Wood KJ, Goto R (2010) Mechanisms of rejection: current perspectives. Transplantation 93: 1–10. 10.1097/TP.0b013e31823cab44 22138818

[2] Schonder KS (2011) Pharmacology of immunosuppressive medications in solid organ transplantation. Crit Care Nurs Clin North Am 23: 405–423. 22054818 10.1016/j.ccell.2011.06.001

[3] Mendieta-Zerón H (2011) Developing immunologic tolerance for transplantation at the fetal stage. Immunotherapy 3: 1499–1512. 22091685 10.2217/imt.11.142

[4] Morelli AE (2006) The immune regulatory effect of apoptotic cells and exosomes on dendritic cells: its impact on transplantation. Am J Transplant 6: 254–261. 16426309 10.1111/j.1600-6143.2005.01197.x

[5] van Kooten C, Lombardi G, Gelderman KA, Sagoo P, Buckland M, et al (2011) Dendritic cells as a tool to induce transplantation tolerance: obstacles and opportunities. Transplantation 91: 2–7. 21452405 10.1097/tp.0b013e31820263b3

[6] Chaput N, Théry C (2011) Exosomes: immune properties and potential clinical implementations. Semin Immunopathol 33: 419–440. 21174094 10.1007/s00281-010-0233-9

[7] Admyre C, Johansson SM, Paulie S, Gabrielsson S (2006) Direct exosome stimulation of peripheral human T cells detected by ELISPOT. Eur J Immunol 36: 1772–1781. 16761310 10.1002/eji.200535615

[8] Montecalvo A, Shufesky WJ, Stolz DB, Sullivan MG, Wang Z, et al (2008) Exosomes as a short-range mechanism to spread alloantigen between dendritic cells during T cell allorecognition. J Immunol 180: 3081–3090. 18292531 10.4049/jimmunol.180.5.3081

[9] Segura E, Nicco C, Lombard B, Véron P, Raposo G, et al (2005) ICAM-1 on exosomes from mature dendritic cells is critical for efficient naive T cell priming. Blood 106: 216–223. 15790784 10.1182/blood-2005-01-0220

[10] Bianco NR, Kim SH, Ruffner MA, Robbins PD (2009) Therapeutic effect of exosomes from indoleamine 2, 3-dioxygenase-positive dendritic cells in collagen-induced arthritis and delayed-type hypersensitivity disease models. Arthritis Rheum 60: 380–389. 19180475 10.1002/art.24229 PMC3491653

[11] Kim SH, Bianco NR, Shufesky WJ, Morelli AE, Robbins PD (2007) Effective treatment of inflammatory disease models with exosomes derived from dendritic cells genetically modified to express IL-4. J Immunol 179: 2242–2249. 17675485 10.4049/jimmunol.179.4.2242

[12] Yang X, Meng S, Jiang H, Chen T, Wu W (2010) Exosomes derived from interleukin-10-treated dendritic cells can inhibit trinitrobenzene sulfonic acid-induced rat colitis. Scand J Gastroenterol 45: 1168–1177. 20469967 10.3109/00365521.2010.490596

[13] Yang X, Meng S, Jiang H, Zhu C, Wu W (2011) Exosomes derived from immature bone marrow dendritic cells induce tolerogenicity of intestinal transplantation in rats. Surg Res 171: 826–832. 10.1016/j.jss.2010.05.021 20828738

[14] Pêche H, Heslan M, Usal C, Amigorena S, Cuturi MC (2003) Presentation of donor major histocompatibility complex antigens by bone marrow dendritic cell-derived exosomes modulates allograft rejection. Transplantation 76: 1503–1510. 14657694 10.1097/01.TP.0000092494.75313.38

[15] Pêche H, Trinite B, Martinet B, Cuturi MC (2005) Prolongation of heart allograft survival by immature dendritic cells generated from recipient type bone marrow progenitors. Am J Transplant 5: 255–267. 15643985 10.1111/j.1600-6143.2004.00683.x

[16] Lutz MB, Kukutsch N, Ogilvie AL, Rössner S, Koch F, et al (1999) An advanced culture method for generating large quantities of highly pure dendritic cells from mouse bone marrow. J Immunol Methods 223: 77–92. 10037236 10.1016/s0022-1759(98)00204-x

[17] Lamparski HG, Metha-Damani A, Yao JY, Patel S, Hsu DH, et al (2002) Production and characterization of clinical grade exosomes derived from dendritic cells. J Immunol Methods 270: 211–226. 12379326 10.1016/s0022-1759(02)00330-7

[18] Hackstein H, Taner T, Zahorchak AF, Morelli AE, Logar AJ, et al (2003) Rapamycin inhibits IL-4-induced dendritic cell maturation in vitro and dendritic cell mobilization and function in vivo. Blood 101: 4457–4463. 12531798 10.1182/blood-2002-11-3370

[19] Taner T, Hackstein H, Wang Z, Morelli AE, Thomson AW (2005) Rapamycin-treated, alloantigen-pulsed host dendritic cells induce Ag-specific T cell regulation and prolong graft survival. Am J Transplant 5: 228–236. 15643982 10.1046/j.1600-6143.2004.00673.x

[20] Turnquist HR, Cardinal J, Macedo C, Rosborough BR, Sumpter TL, et al (2010) mTOR and GSK-3 shape the CD4+ T-cell stimulatory and differentiation capacity of myeloid DCs after exposure to LPS. Blood 115: 4758–4769. 20335217 10.1182/blood-2009-10-251488 PMC2890188

[21] Pliszczynski J, Kahan BD (2011) Better actual 10-year renal transplant outcomes of 80% reduced cyclosporine exposure with sirolimus base therapy compared with full cyclosporine exposure without or with concomittant sirolimus treatment [J]. Transplant Proc 43: 3657–3668. 22172822 10.1016/j.transproceed.2011.10.052

[22] Inaba K, Inaba M, Romani N, et al (1992) Generation of large numbers of dendritic cells from mouse bone marrow cultures supplemented with granulocytermacrophage colonystimulating factor. J Exp Med 176: 1693–1702. 1460426 10.1084/jem.176.6.1693 PMC2119469

[23] Sallusto F, Lanzavecchia A (1994) Efficient presentation of soluble antigen by cultured human dendritic cells is maintained by granulocytermacrophage colony-stimulating factor plus interleukin 4 and downregulated by tumor necrosis factor alpha. J Exp Med 179: 1109–1118. 8145033 10.1084/jem.179.4.1109 PMC2191432

[24] Hume PS, He J, Haskins K, Anseth KS (2012) Strategies to reduce dendritic cell activation through functional biomaterial design. Biomaterials 33: 3615–3625. 22361099 10.1016/j.biomaterials.2012.02.009 PMC3318919

[25] Morse MA, Garst J, Osada T, Khan S, Hobeika A, et al (2005) A phase I study of dexosome immunotherapy in patients with advanced non-small cell lung cancer. J Transl Med 3: 9–9. 15723705 10.1186/1479-5876-3-9 PMC551593

[26] Kim SH, Lechman ER, Bianco N, Menon R, Keravala A, et al (2005) Exosomes derived from IL-10-treated dendritic cells can suppress inflammation and collagen-induced arthritis. J Immunol 174: 6440–6448. 15879146 10.4049/jimmunol.174.10.6440

[27] Battaglia M, Stabilini A, Roncarolo MG (2005) Rapamycin selectively expands CD4+CD25+FoxP3+ regulatory T cells. Blood 105: 4743–4748. 15746082 10.1182/blood-2004-10-3932

[28] Monti P, Scirpoli M, Maffi P, Piemonti L, Secchi A, et al (2008) Rapamycin monotherapy in patients with type 1 diabetes modifies CD4+CD25+FOXP3+ regulatory T-cells. Diabetes 57: 2341–2347. 18559659 10.2337/db08-0138 PMC2518485

[29] Morelli AE, Larregina AT, Shufesky WJ, Sullivan ML, Stolz DB, et al (2004) Endocytosis, intracellular sorting, and processing of exosomes by dendritic cells. Blood 104: 3257–3266. 15284116 10.1182/blood-2004-03-0824

[30] Wang GJ, Liu Y, Qin A, Shah SV, Deng ZB, et al (2008) Thymus exosomes-like particles induce regulatory T cells. J Immunol 181: 5242–5248. 18832678 10.4049/jimmunol.181.8.5242 PMC4319673

[31] Cai Z, Zhang W, Yang F, Yu L, Yu Z, et al (2012) Immunosuppressive exosomes from TGF-β1 gene-modified dendritic cells attenuate Th17-mediated inflammatory autoimmune disease by inducing regulatory T cells. Cell Res 22: 607–610. 22157651 10.1038/cr.2011.196 PMC3292292

[32] Muthana M, Fairburn B, Mirza S, Slack LK, Pockley AG (2004) Systematic evaluation of the conditions required for the generation of immature rat bone marrow-derived dendritic cells and their phenotypic and functional characterization. J Immunol Methods 294(1–2): 165–179. 15604025 10.1016/j.jim.2004.09.006

[33] Niimi M (2001) The technique for heterotopic cardiac transplantation in mice: experience of 3000 operations by one surgeon. J Heart Lung Transplant 20: 1123–1128. 11595568 10.1016/s1053-2498(01)00309-6

[34] Chen ZH (1991) A technique of cervical heterotopic heart transplantation in mice. Transplantation 52: 1099–1101. 1750075 10.1097/00007890-199112000-00035

